# Cytoscape: the network visualization tool for GenomeSpace workflows

**DOI:** 10.12688/f1000research.4492.2

**Published:** 2014-08-26

**Authors:** Barry Demchak, Tim Hull, Michael Reich, Ted Liefeld, Michael Smoot, Trey Ideker, Jill P. Mesirov

**Affiliations:** 1 Department of Medicine, University of California, San Diego, La Jolla, 92093-0688, USA; 2 Broad Institute of Massachusetts Institute of Technology and Harvard, Cambridge, 02142, USA; 3 Qualcomm, Inc, San Diego, 92121, USA

## Abstract

Modern genomic analysis often requires workflows incorporating multiple best-of-breed tools. GenomeSpace is a web-based visual workbench that combines a selection of these tools with mechanisms that create data flows between them. One such tool is Cytoscape 3, a popular application that enables analysis and visualization of graph-oriented genomic networks. As Cytoscape runs on the desktop, and not in a web browser, integrating it into GenomeSpace required special care in creating a seamless user experience and enabling appropriate data flows. In this paper, we present the design and operation of the Cytoscape GenomeSpace app, which accomplishes this integration, thereby providing critical analysis and visualization functionality for GenomeSpace users. It has been downloaded over 850 times since the release of its first version in September, 2013.

## Introduction

GenomeSpace is a web-based application ( http://genomespace.org) that provides a workspace environment for executing biologic analysis workflows involving genomic data. It hosts a variety of third party tools that it can launch to perform queries of public genomic databases, customized analyses, and customized visualization and publishing. The data and results from a given tool are stored by GenomeSpace as private, public, or shared files that are made available to other tools in the workspace. Cytoscape is a standalone desktop application that enables users to analyze, visualize, and publish complex networks – with its GenomeSpace app (“the app”, http://apps.cytoscape.org/apps/genomespace), it doubles as a tool available to GenomeSpace workflows. This paper describes Cytoscape’s GenomeSpace app, which links GenomeSpace and Cytoscape, thereby enabling data to flow between Cytoscape and other GenomeSpace tools. It focuses on critical design issues for the app, and gives a brief app demonstration highlighting the resulting user interface and data transfer functionality.

While there exists a number of workflow engines that can perform biological analyses (e.g., Taverna ^1^, BioKepler ^2^, and Galaxy ^3^), GenomeSpace distinguishes itself by combining a collaboration-oriented file system (incorporating sharing and stored metadata), a robust and user-extensible spectrum of genomic tools, and a library of recipes demonstrating best practices for the orchestration of GenomeSpace tools to achieve common and important bioinformatic results. While some tools implement specific and constrained functionality (e.g., ISAcreator ^4^), others are complex and rich applications (e.g., GenePattern ^5^, Gitools ^6^, and Cistrome ^7^), and yet others are fully featured workflow management systems themselves (e.g., Galaxy and Sage Synapse ^8^). By tying these features together, GenomeSpace provides a comprehensive and effective environment for genomic research.

GenomeSpace offers a short list of tools that enable network visualization and analysis, including Cytoscape, Genomica ( http://genomica.weizmann.ac.il/) (for module network trees), Gitools ^6^ (for heat maps), and IGV ^9^ (for sequencing data). Cytoscape distinguishes itself by being graph-oriented and delivering rich filtering, layout, and visual style features backed up by an extensive collection of third-party apps ^10^, including pathway analysis, data integration, GO annotation, and more.

To launch a tool, GenomeSpace opens a new browser page using a URL specific to the tool. Paradoxically, Java-based tools (such as Cytoscape) run directly on the user’s workstation instead of within a browser. In this paper, we describe a specialized launch strategy that addresses this. We also describe Cytoscape’s GenomeSpace app, how its user interface adds GenomeSpace functionality within Cytoscape, and how it uses GenomeSpace’s Client Development Kit (CDK) to access the GenomeSpace file system to read input files or write result files.

Note that GenomeSpace supports two Cytoscape tools it calls “Cytoscape” and “Cytoscape 3”. Its “Cytoscape” refers to the deprecated Cytoscape version 2, and its “Cytoscape 3” refers to Cytoscape version 3, which is the currently released version ( http://cytoscape.org). Within this paper, we discuss only the “Cytoscape 3” tool, and refer to it simply as “Cytoscape”.

## Implementation

The Cytoscape support for GenomeSpace exists in three parts: the launch support, Cytoscape’s GenomeSpace app, and Cytoscape itself. This section describes how the launch support and app work, and leaves the operation of Cytoscape to the Results section below. Technical details of Cytoscape internal organization, construction, APIs, data structures, and general conventions can be found in the Cytoscape App Developer wiki ( http://wiki.cytoscape.org/Cytoscape_3/AppDeveloper).

### Launch

**Figure 1.**
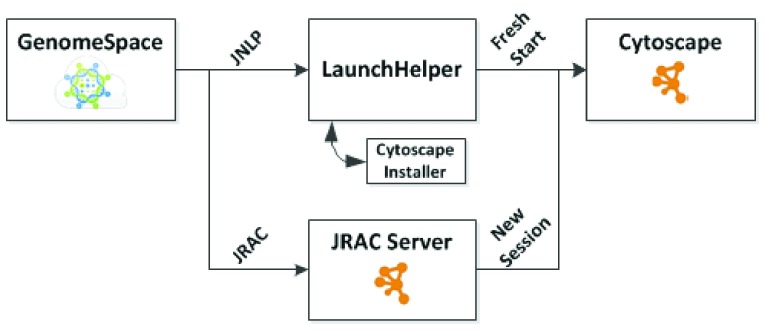
Launching Cytoscape from GenomeSpace.

To launch a tool, a GenomeSpace user left-clicks on the corresponding toolbar icon, which activates the tool via the tool’s URL. For Cytoscape, the URL references a launch descriptor file that adheres to the Java Network Launch Protocol (JNLP) ^11^ and resides on the Cytoscape web site. Web browsers process a JNLP file URL by starting a specialized launcher that downloads a Java application named in the JNLP file, then executes it on the user’s workstation. In Cytoscape’s case, we created a dynamic JNLP file (as a PHP script that delivers a JNLP file) that executes a small LaunchHelper Java application (see Figure 1). GenomeSpace constructs the JNLP URL to contain a GenomeSpace file descriptor as a parameter, and the PHP script extracts it and defines it as a parameter to the LaunchHelper (see Supplementary Data, particularly as the value used for the SomeGenomeSpaceFileID in the gs.url argument).

The LaunchHelper tests for the presence of Cytoscape and Cytoscape’s GenomeSpace app, and installs them if they are not present. Because LaunchHelper is itself a Java application, it is able to download the Cytoscape installer appropriate for the user’s workstation and the GenomeSpace app while maintaining an interactive user interface, including appropriate installation dialog boxes (as JOptionPane) and progress bars (as ProgressMonitorInputStream).

Note that before attempting to launch Cytoscape, GenomeSpace attempts to determine if Cytoscape is already running by using the JRAC protocol ( http://code.google.com/p/jrac) – if it is, Cytoscape simply starts a new session.

### GenomeSpace app

The GenomeSpace app manages the relationship between Cytoscape and GenomeSpace once Cytoscape is running. In addition to responding to a JRAC request (above), it augments the Cytoscape user interface to allow the user to directly access the GenomeSpace file system and tools.

### Building app menus

The app exposes a number of GenomeSpace functions as menu items under Cytoscape’s File and Apps menus. This enables GenomeSpace login, opening and saving GenomeSpace sessions, launching GenomeSpace tools, and importing and exporting networks and tables as GenomeSpace files. Networks can be exported in .sif, .cyjs, .nnf, PSI-MI, and .xgmml formats. While some menu items are positioned within Cytoscape’s top-level menus (e.g., session open and save menu items in the File menu), others are positioned in submenus within Cytoscape menu items (e.g., importing a network under File | Import | Network). For nice effect, each menu item identifies itself with a distinctive GenomeSpace logo and uses menu gravity to place itself consistently relative to existing Cytoscape menu items. (It uses setPreferredMenu to add the menu, setMenuGravity to position it, and putValue to set the small icon).

### GenomeSpace communication strategy

To implement these menu items, the app communicates with GenomeSpace via the GenomeSpace CDK ( http://www.genomespace.org/support/api/cdk), a proxy interface to GenomeSpace carried over an SSL Internet connection. The CDK enables GenomeSpace session management, tool discovery, user authentication, file system listing, and file upload and download. As with other apps, the app’s cyActivator initializes the app state, including gaining references to the standard Cy objects: application, network, view, and table managers. It also initializes basic CDK-related state (e.g., the GenomeSpaceContext root context).

### GenomeSpace file I/O

In addition to communicating with GenomeSpace, the CDK displays key GenomeSpace-related dialog boxes, including the login dialog and a file chooser for import and export functions. The app uses file choosers to identify an import (or export) file (referenced by metadata), but then executes the operation using a combination of CDK download/upload functions and Cytoscape task manager and monitor functions.

For example, given metadata for a network file to import, the app creates a Cytoscape task iterator that downloads the network to a temp file, loads the network into Cytoscape, and then deletes the temp file as shown in the Supplementary Data. DownloadFileFromGenomeSpaceTask calls CDK to perform the download, and loadNetworkFileTaskFactory adds the network to the Cytoscape data model. Because the app orchestrates the download using Cytoscape’s task manager, its progress is automatically tracked and reported by Cytoscape’s task monitor.

## Results

The GenomeSpace app enables Cytoscape to act as a tool in a workflow executed within the GenomeSpace web application. From the Cytoscape perspective, genomic data can come from numerous sources and can be rendered to numerous destinations, where the GenomeSpace file system can be a source, a destination, or both. To facilitate this, GenomeSpace also allows the user to identify a GenomeSpace-stored Cytoscape session file (.cys) while launching Cytoscape, thereby facilitating a seamless tool launch.

Additionally, the app adds menu items to Cytoscape to enable loading or saving a Cytoscape session, a network, or node or edge attributes in the GenomeSpace file system from within Cytoscape. Each menu item enables the user to navigate within the file system using a chooser.

Note that the GenomeSpace app makes the GenomeSpace file system available to Cytoscape even without the user first executing the GenomeSpace web application. In this case, Cytoscape enables the user to log into GenomeSpace within Cytoscape, and then use the app-injected menus to access workflow-related data files.

### Demonstration

As a demonstration of typical Cytoscape usage, we show how to use Cytoscape to integrate gene expression data with a pre-defined genomic network, where both the network and gene expression data reside in the GenomeSpace file system. The network was created in a prior Cytoscape session and stored in the GenomeSpace file system so it could be shared as a template with collaborators and integrated with various gene expression datasets produced during an ongoing study. The test network is a portion of the BioGrid H. sapiens network. The gene expression data represents the output of some previously executed GenomeSpace tool, such as Galaxy.

**Figure 2.**
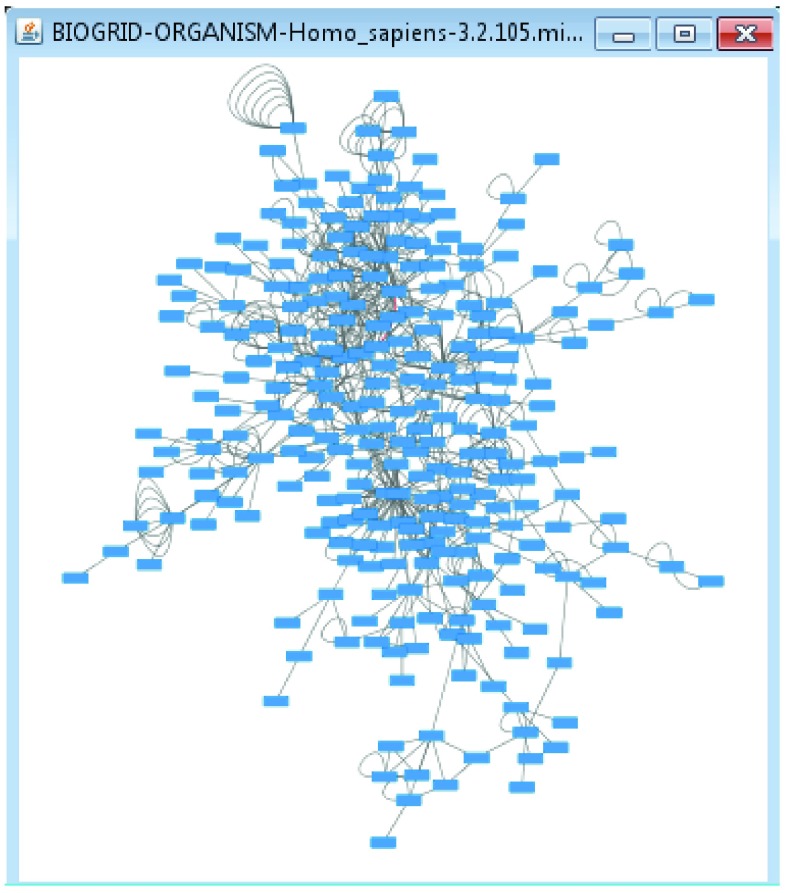
Template H. sapiens before gene expression data.

To load the template network into Cytoscape, use a web browser on a workstation having at least 6GB RAM. Log into GenomeSpace.org – you can easily create a user ID if you don’t have one. Right-click on the “Cytoscape 3” tool, and choose the Launch on File menu item. Choose the Cytoscape session file (.cys) containing the H. sapiens network template by browsing the file system to /bdemchak/F1000Example, then dragging it to the launch box and clicking Launch. Once Cytoscape has started and the network has loaded, you will see a small network in a view window ( Figure 2).

To load a gene expression dataset, choose Cytoscape’s File | Import | Table | GenomeSpace menu item, and use the chooser to select the enrichment data in the /bdemchak/F1000Example folder. Cytoscape will display an Import Columns from Table dialog – when you click on OK, note that the enrichment data (including values in the HSC1_1 column) has been added to the node table.

**Figure 3.**
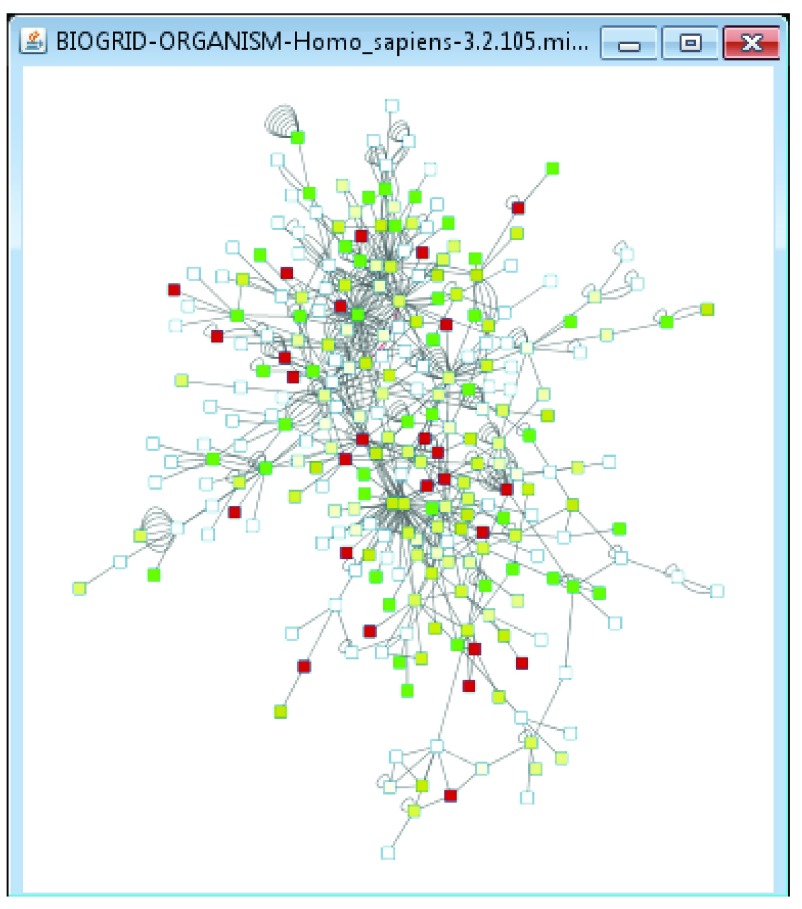
Template H. sapiens after gene expression data.

Finally, to use the gene expression data to color the network, select Cytoscape’s Style tab and choose the RedYellowGreen style from the style dropdown. Nodes are colored by their associated HSC1_1 value, and nodes having no HSC1_1 value are left white ( Figure 3). This style is part of the Cytoscape session file loaded for this example, and illustrates that all Cytoscape functionality is available in Cytoscape operating as a GenomeSpace tool.

Refer to the Cytoscape manual ( http://wiki.cytoscape.org/Cytoscape_3/UserManual) and tutorials ( http://tutorials.cytoscape.org) for a more detailed treatment of Cytoscape workflows.

## Conclusions

The GenomeSpace app shows how Cytoscape can be used as a plugin to a web application as part of a larger workflow, and then how to integrate external services into Cytoscape’s workflow.

## Software availability

Software available from: http://apps.cytoscape.org/apps/genomespace

Latest source code:

https://github.com/idekerlab/genomespace-cytoscape-weblaunch

https://github.com/idekerlab/genomespace-cytoscape

Source code as at the time of publication: https://github.com/F1000Research/genomespace-cytoscape-weblaunch/releases/tag/v1.0

https://github.com/F1000Research/genomespace-cytoscape/releases/tag/V1.0

Archived source code as at the time of publication: http://www.dx.doi.org/10.5281/zenodo.10441 ^12^

http://www.dx.doi.org/10.5281/zenodo.10536 ^13^

License: Lesser GNU Public License v2.1
