# Supplementary material for: Cytoscape: the network visualization tool for GenomeSpace workflows
Source: F1000Res. 2014 Aug 26;3:151. Originally published 2014 Jul 1. [Version 2] doi: 10.12688/f1000research.4492.2 (PMC4133763; doi:10.12688/f1000research.4492.2)
Supplement: Peer review history

# Peer review: Referee response for version 1

**DOI:** 10.5256/f1000research.4805.r5449

**Published:** 2014-08-04

**Authors:** Jan Aerts

**Affiliations:** 1 Bioinformatics, KU Leuven, Leuven, Belgium

In the paper "Cytoscape: the network visualization tool for GenomeSpace workflows", Demchak *et al. *describe the design, implementation and operation of an interaction layer between Cytoscape and GenomeSpace.

Unfortunately, I was not able to run the example described in the manuscript, due to a "unable to find the document" error.

Overall, the manuscript gives a fair description both of the technology used for integrating Cytoscape into GenomeSpace and its use. This however does mean that the actual focus is not completely clear. If the message is "there is a new tool accessible from GenomeSpace" which would be aimed at the end-user, then terms like "JRAC" and "cyActivator" cannot be expected to be understood without further explanation. Care should be given to explain these sufficiently enough for lay-people. On the other hand, the message could also be "here is how to integrate a web-tool with a desktop application", in which case the technical part might be described a bit more in depth.

I expect this paper and the bridge between Cytoscape and GenomeSpace will be of considerable interest to the community.

Minor textual remarks:

- In the abstract, there is an extra "it" in "It has been downloaded it over 850 times".
- Hyphenation has gone awry in many cases. Although this is not important for the contents of the manuscript, it does start to bother the reader after a while. These include (but are not limited to): "GenomeS-pace", "submen-us", "grav-ity", "Genom-eSpace", "Genom-eSpaceContext", "attrib-utes", and "Bi-oGrid".

I have read this submission. I believe that I have an appropriate level of expertise to confirm that it is of an acceptable scientific standard.

# Peer review: Referee response for version 1

**DOI:** 10.5256/f1000research.4805.r5651

**Published:** 2014-08-04

**Authors:** Gabriela Bindea, Bernhard Mlecnik

**Affiliations:** 1 Laboratory of Integrative Cancer Immunology, Cordelier Research Center, Paris, France

The paper “Cytoscape: the network visualization tool for GenomeSpace workflows” describes the GenomeSpace app, a tool that facilitates data analysis in Cytoscape for GenomeSpace users. This is a nice try to bridge two communities: cytoscapers & genomespacers.

The resources and tools included in GenomeSpace are well presented, and the important data sharing possibility is underlined. At the same time, the extensive app collection of Cytoscape with its multiple analysis possibilities is very short mentioned. This could be maybe extended so that users of GenomeSpace could use Cytoscape not only to visualize their results obtained in GenomeSpace, but also to analyze their data with Cytoscape apps. But this will be probably the topic of another paper.

For running the app, the computer should have enough memory, as mentioned in the article. Some GUI adjustments should be done for the different OS, for instance in Ubuntu the login dialog size should be increased, the buttons are not entirely visible at the moment. The new functionality is nicely underlined with icons, but is scattered throughout the menu. To increase the usability/visibility, after the login it would be good e.g. to add also the import/export buttons into the tool bar in top.

The app performs well. It remains to the users of both communities to discover the multiple possibilities that this app offers.

We have read this submission. We believe that we have an appropriate level of expertise to confirm that it is of an acceptable scientific standard.

# Peer review: Referee response for version 1

**DOI:** 10.5256/f1000research.4805.r5291

**Published:** 2014-07-30

**Authors:** Aris Floratos

**Affiliations:** 1 Department of Systems Biology, Columbia University, New York, NY, USA

**Language/spelling:**

The language is acceptable but would benefit from a more careful reading/editing. Some examples where improvements are possible are:

- “Javabased” should be corrected to “Java-based”.
- In the sentence that begins “Web browsers are defined to process a JNLP file URL by starting a specialized launcher…”, the portion that reads “are defined to” is extraneous.
- In the sentence that reads “Paradoxically, Javabased tools (such as Cytoscape) run directly on the user’s workstation instead of within a browser”, it is not clear what is paradoxical about the described execution protocol.  The word “paradoxically” should/could be dropped or maybe be replaced by a more appropriate adverb.

**Comments on specific sections:**

- *Launch*: there is a sentence that reads “GenomeSpace constructs the JNLP URL to contain a GenomeSpace file descriptor as a parameter, and the PHP script extracts it and defines it as a parameter to the LaunchHelper (see Supplementary Data).” It would add to clarity to explain what the file referenced is.
- *GenomeSpace app:* This section appears written with the assumption that the reader is already familiar with the Cytoscape app framework, essentially no background is provided.
- *GenomeSpace communication strategy*: Same comment as above: the article refers to Cytoscape-specific programming constructs without introducing them or providing relevant references for disambiguation. E.g., in the sentence “As with other apps, the app’s cyActivator initializes the app state, including gaining references to the standard Cy objects: application, network, view, and table managers”, terms like “cyActivator” and “Cy objects” are used without prior definition/explanation. The same issue exists with GenomeSpace-specific objects, e.g., the sentence “It also initializes basic CDK-related state (e.g., the GenomeSpaceContext root context)”.
- *Demonstration*: It would be helpful to describe what the columns of the input data file EnrichmentData.dat represent.
- *Demonstration*: The demonstration would benefit from a more detailed analysis workflow. E.g., a gene expression data set and a class file can be processed by GenePattern (or some other GenomeSpace tool) to generate a list of differentially expressed genes for some reasonable pair of case/control phenotypes and then feed the resulting file into Cytoscape. This could also help demonstrate the value proposition of GenomeSpace, i.e., providing the ability to seamlessly use multiple tools within an integrated environment. The authors may also want to briefly discuss why people are often interested in visualizing enriched/differentially-expressed genes in the context of a network, this would help the less-initiated better appreciate the value of having Cytoscape available as a GenomeSpace tool.

**Overall Impression:**

This is an informative article, describing how a specific tool (Cytoscape) has been integrated into an innovative framework (GenomeSpace) that enables seamless access to multiple bioinformatics tools. The main concern is overall readability in the absence of sufficient background on the Cytoscape app framework, to put the implementation of the GenomeSpace app in proper context. The other issues are minor and can be easily addressed.

I have read this submission. I believe that I have an appropriate level of expertise to confirm that it is of an acceptable scientific standard.
